# Core outcome sets for use in effectiveness trials involving people with bipolar and schizophrenia in a community-based setting (PARTNERS2): study protocol for the development of two core outcome sets

**DOI:** 10.1186/s13063-015-0553-0

**Published:** 2015-02-12

**Authors:** Thomas Keeley, Humera Khan, Vanessa Pinfold, Paula Williamson, Jonathan Mathers, Linda Davies, Ruth Sayers, Elizabeth England, Siobhan Reilly, Richard Byng, Linda Gask, Mike Clark, Peter Huxley, Peter Lewis, Maximillian Birchwood, Melanie Calvert

**Affiliations:** School of Health and Population Sciences, University of Birmingham, Birmingham, B15 2TT UK; The McPin Foundation, London, SE1 OEH UK; Institute of Translational Medicine, University of Liverpool, Liverpool, L69 3BX UK; Centre for Health Economics, University of Manchester, Manchester, M13 9PL UK; Division of Health Research, Lancaster University, Lancaster, LA1 4YG UK; Centre for Clinical Trials and Health Research, Plymouth University, Plymouth, PL4 8AA UK; Institute of Population Health, University of Manchester, Manchester, M13 9PL UK; NIHR School for Social Care Research, London School of Economics and Political Science, London, WC2A 2AE UK; Centre for Mental Health and Society, Bangor University, Bangor, LL57 2DG UK; Birmingham and Solihull Mental Health Foundation Trust, Birmingham, B1 3RB UK; Mental Health and Wellbeing, University of Warwick, Warwick, CV4 7AL UK

## Abstract

**Background:**

In the general population the prevalence of bipolar and schizophrenia is 0.24% and 1.4% respectively. People with schizophrenia and bipolar disorder have a significantly reduced life expectancy, increased rates of unemployment and a fear of stigma leading to reduced self-confidence. A core outcome set is a standardised collection of items that should be reported in all controlled trials within a research area. There are currently no core outcome sets available for use in effectiveness trials involving bipolar or schizophrenia service users managed in a community setting.

**Methods:**

A three-step approach is to be used to concurrently develop two core outcome sets, one for bipolar and one for schizophrenia. First, a comprehensive list of outcomes will be compiled through qualitative research and systematic searching of trial databases. Focus groups and one-to-one interviews will be completed with service users, carers and healthcare professionals. Second, a Delphi study will be used to reduce the lists to a core set. The three-round Delphi study will ask service users to score the outcome list for relevance. In round two stakeholders will only see the results of their group, while in round three stakeholders will see the results of all stakeholder group by stakeholder group. Third, a consensus meeting with stakeholders will be used to confirm outcomes to be included in the core set. Following the development of the core set a systematic literature review of existing measures will allow recommendations for how the core outcomes should be measured and a stated preference survey will explore the strength of people’s preferences and estimate weights for the outcomes that comprise the core set.

**Discussion:**

A core outcome set represents the minimum measurement requirement for a research area. We aim to develop core outcome sets for use in research involving service users with schizophrenia or bipolar managed in a community setting. This will inform the wider PARTNERS2 study aims and objectives of developing an innovative primary care-based model of collaborative care for people with a diagnosis of bipolar or schizophrenia.

## Background

Mental illness is the single largest cause of disability in the UK, contributing to 22.8% of the total burden of disease [1]. An evaluation of primary care service provision for severe mental illness (SMI) indicates that 94% of service users had a recorded diagnosis of schizophrenia or bipolar disorder [2]. In the general population the prevalence of schizophrenia is up to 1.4% and for bipolar I disorder it is 0.24% [3]. People with schizophrenia and bipolar disorder have a significantly reduced life expectancy, increased rates of unemployment and experience of stigma leading to reduced self-confidence and self-esteem [4,5]. The service cost alone of treating these diseases is estimated at £3.8 billion and expected to rise to £6.3 billion per year [6,7].

Randomised controlled trials (RCTs) can provide robust evidence to inform mental health and social care. Outcomes assessed in trials can help inform decisions regarding both individual care and policy formation at local, regional or national levels. To do this outcomes need to be relevant and of value to stakeholders, such as the services users, family, carers, health and social care professionals and decision makers.

The use of numerous trial outcomes within the same research area can cause challenges. The most accessed and cited Cochrane reviews of 2009 all reported problems with heterogeneity of outcomes [8,9]. Within mental health research, trials frequently use a broad range of outcome measures. A recent survey of 10,000 RCTs in schizophrenia reported that 2,194 different measurements were used, with a new outcome being reported every fifth trial [10]. This has the potential to reduce the ability to synthesise results. Furthermore, many of the measures used in these trials have been selected by researchers and clinicians and may not reflect outcomes^a^ that are relevant to all stakeholders.

The use of numerous outcome measures within a controlled trial can also result in reporting bias. RCTs should specify *a priori* the primary and secondary outcome measures to be used to test a study hypothesis [11-13]. However, reporting bias occurs when the authors report a subsection of the outcome measures, based on the significance of the findings [14]. Reporting bias has been shown to be a widespread phenomenon in medical literature generally [15,16], as well as in mental health specifically [17].

These issues can be addressed through the use of a core outcome set with input from a range of stakeholders including service users with lived experience of SMI and carers. A core outcome set is a standardised collection of items that should be reported in all controlled trials within a research area [9]. It represents the minimum outcomes that should be measured and reported [18]. Trialists are not restricted to these measures and can use additional measures to those in the core set. However, the core set marks the basic requirement of what outcomes need to be measured and reported.

Core outcome sets for effectiveness trials involving service users with bipolar or schizophrenia, managed in a community setting, have the potential to reduce reporting bias and facilitate evidence synthesis. The assessment of similarities in outcomes between the two sets may allow the identification of common outcomes for use in SMI trials including both groups. Community setting refers to care or support received while living in the community (i.e. not in hospital as an in-patient). A search of the COMET database showed no core outcome sets are currently available for use in controlled trials recruiting adult participants with bipolar or schizophrenia, managed in a community setting.

The PARTNERS2 study has been funded through the NIHR under its Programme Grants for Applied Research programme (grant reference no. RP-PG-0611-20004). It aims to help primary care and community-based mental health services work more closely and efficiently together through developing and trialling an innovative primary care-based model of collaborative care for people with a diagnosis of bipolar and schizophrenia. Work commenced in March 2014 and is scheduled for completion in 2019.

## Aims and objectives

### Aims

**Figure 1:**
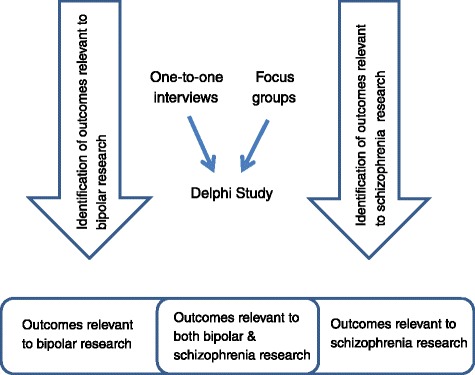
**Identification of a core outcome sets for schizophrenia and bipolar.**

The aim of this study is to develop two individual core outcome sets for schizophrenia and bipolar for use in SMI trials involving adult service users recruited from a community setting. In addition, we will compare the two core outcome sets and identify common outcomes to be assessed in SMI trials including both groups (see Figure 1).

### Objectives

The specific objectives are threefold. First, to identify a comprehensive list of outcomes that are relevant for trials involving schizophrenia and bipolar disorder in a community setting, to develop two core outcome sets based on this comprehensive list and, where possible, to identify common outcomes across core outcome sets. The second is to identify potential reliable, valid and responsive measures that could be used to assess the outcomes included in the core sets. A further objective is to estimate the strength of people’s preferences and weights for key outcomes/items included in the patient-reported outcome measures (PROMs) that comprise the core sets.

## Methods

### Overview

A three-step approach to concurrently developing each of the core outcome sets will be used [9,19]. First, a comprehensive list of outcomes relevant to stakeholders will be compiled through qualitative research and through a review of outcomes reported in existing trials. Focus groups and one-to-one interviews with key stakeholders will be used. Second, the comprehensive list will be reduced to a core outcome set through Delphi methodology. Third, a consensus meeting with stakeholders will confirm the outcomes to be included in the core sets.

Two additional steps will then be completed. A systematic literature review of existing measures and a stakeholder discussion will allow recommendations as to how the core outcomes should be measured. Finally, a stated preference survey will explore the strength of people’s preferences and estimate weights for key outcomes/items included in the PROMs that comprise the core sets.

Ethical approval for the study has been sought and granted from the National Research Ethics Service (NRES) West Midlands – Edgbaston (reference no. 14/WM/0052).

### Step 1: Qualitative research

The opinions of key stakeholders will be sought using focus groups and one-to-one interviews. The qualitative methods will differ between stakeholder groups due to methodological and practical considerations, such as time pressures of healthcare professionals.

#### Focus groups and one-to-one interviews with service users and carers

##### Participants

People with schizophrenia or bipolar who use community services to support their health and well-being and their family members and carers will be purposively sampled by age, gender, condition and geographical location. To be eligible for inclusion in a focus group or interview service users must: self-identify as having a lifetime or current clinical diagnosis of schizophrenia or bipolar; be receiving treatment in a community setting; be over 18 and under 65 years old and speak English. Carers must be a self-reported carer of a person who meets the service user criteria and speaks English.

##### Recruitment

Recruitment will occur through NHS Trust clinics and via the third sector organisations, such as MIND, National Survivor User Network (NSUN), Rethink Mental Illness, Bipolar UK and Carers in Partnership. Initial contact will be via email, post and leaflets and posters displayed in NHS clinics and third sector organisations. Interested participants will be advised to contact the study team by post, email or phone. The study team will check the eligibility and identify potential dates for participation. At this point the research will be described in greater detail and potential participants will be given the opportunity to ask questions. An invitation letter and an information sheet will be sent to eligible participants that wish to participate. A reminder phone call will be scheduled, or letter will be sent, in the week prior to the focus group. For those participants preferring to take part in one-to-one interviews, these will be arranged and a reminder phone call scheduled. For both focus groups and one-to-one interviews informed consent will be taken prior to starting data collection.

The number of service user and carer participants recruited to the study is dependent on a number of factors: the proportion of the data gathered through focus groups as opposed to one-to-one interviews^b^, the size of the focus groups that are held and the point at which data saturation is achieved. It is anticipated that roughly 24 service users (12 with each diagnosis) and up to 10 carers will be recruited to the qualitative stage of the work.

##### Methodology

Focus groups and one-to-one interviews will seek to identify clinical, social and psychological outcomes that are important to service users and carers. Participants will be encouraged to discuss the impact that their illness has upon them and their life. They will be asked to explain how treatment or management of their illness affects them. Participants will be encouraged to explore the important short- and long-term impacts of treatment. A topic guide, developed in collaboration with a researcher with lived experience of serious mental illness, will be used as an aide memoire and to add structure to the discussion.

Focus groups will be split by clinical diagnosis resulting in groups of bipolar service users, bipolar carers, schizophrenia service users and schizophrenia carers. This will allow identification of outcomes relevant to bipolar and schizophrenia service users and any common outcomes across diagnoses.

The consent process will be completed on the day of the focus group or interview, before starting the discussion, in order to allow assessment of capacity to participate on that day. Prior to the consent form being signed, participants will be asked if they have read and understood the information provided to them and will have the opportunity to ask any further questions. The audio recording of discussions, the anonymity process and the ability of the participant to withdraw from the focus group at any point will be verbally emphasised at the start.

#### One-to-one interviews with commissioners, policy makers and health and social professionals

##### Participants

Consultant psychiatrists, mental health leads within clinical commissioning groups, general practitioners with a special interest in or professional experience (either clinical or commissioning) of serious mental illness, social workers, community psychiatric nurses, commissioners, policy makers and those with relevant roles in third sector organisations^c^ will be included as participants. Sampling will be stratified and purposive so as to ensure suitable variation of professional groups is achieved.

##### Recruitment

Health and social care professionals will be recruited primarily from West-Midlands and Lancashire. Publically available lists and professional contacts of project investigators will be used to identify potential participants.

Potential participants will be contacted via an email, containing a study information sheet, requesting their participation in the study. A reminder email will be sent 2 weeks after the original email. Upon a positive reply from a potential participant a date will be set for a telephone interview. A consent form will be sent to participants via email upon agreeing to participate. Verbal consent will be taken and recorded at the start of the interview and a request will be made for the participant to return a signed copy of the consent form via post.

The number of health and social care professionals recruited to the study is dependent on adequately sampling each of the professional groups named above and the point at which data saturation is reached. It is anticipated that roughly 20 participants will be recruited to the qualitative stage of the work.

##### One-to-one interview methodology

A semi-structured interview will be undertaken. A topic guide will be used to direct the conversation; however the semi-structured nature of the discussion will allow emergent themes or pertinent points to be explored. Participants will be asked to discuss how bipolar and schizophrenia affects a person’s life, how care and support can improve outcomes that are important to the patient and what outcomes should be assessed in research with these populations. One half of the interview will be given to discussing bipolar and the other half to schizophrenia. The sequence in which the conditions are discussed will be varied. Where a participant has particular expertise in one condition the whole of the interview will be devoted to discussing that condition (this is expected in a minority of cases).

#### Qualitative data analysis

Focus groups and one-to-one interviews will be digitally audio-recorded and transcribed verbatim. Transcripts will be coded using the computer-assisted qualitative data analysis software, such as Dedoose or Nvivo. The first version of the coding structure will be formed during the analysis of the early data and therefore grounded in the data. For the service user and carer data two researchers (one with and one without lived experience) will concurrently analyse all data. For the health and social care professional data the facilitator of the interviews will lead the analysis of data, with 20% checked for accuracy and completion by a researcher with lived experience of mental illness.

An iterative, constant comparative and thematic analysis of the transcripts will be completed [20,21]. The iterative nature of the analysis will allow themes identified in the early focus groups to be explored in greater depths with later groups. The analysis will focus on forming a comprehensive list of outcomes that are important to stakeholders. The structure and length of this list will be dependent on the data.

During the qualitative analysis outcomes identified as relevant to schizophrenia and bipolar service users will be identified separately. Evidence of notable convergence in the outcomes in the two groups may indicate that in addition to the bipolar and schizophrenia core outcome sets, the potential for a joint core outcome set could be considered. Considerable difference in the outcomes at this stage would suggest that this is not a workable possibility.

#### Review of literature

A focussed review of literature and databases will be completed concurrently with the qualitative work in order to ensure a complete set of relevant outcomes is identified. Through this approach the potential of not including outcomes that are important to stakeholders is reduced. The findings of the review will be cross-referenced with the results of the qualitative work to ensure completion.

The literature and databases reviewed will be: Cochrane registers, a recent Cochrane review [22], ongoing trials adopted by the CRNs and trial registries, the outcomes compendium [23] and Outcome Measurement in Mental Health: the views of service users [24]. The following data will be extracted from the literature: basic trial information, investigator names, year of study, study setting, primary outcome, secondary outcomes and measures used.

The results of the qualitative work and the literature review will be merged to form two separate comprehensive lists of outcomes for bipolar and schizophrenia. These will be finalised through discussion with representatives of stakeholder groups, including our Lived Experience Advisory Panel (LEAP). The LEAP will consist of people with personal experience of living with schizophrenia or bipolar (as service users or carers) will be recruited to form Lived Experience Advisory Panels, combining their expertise from lived experience with their insights into research design and delivery. These LEAPs will meet regularly to promote the study locally, problem-solve and ground interpretation. LEAPs will support the development of the core outcome set, through reviewing findings from the qualitative data, helping to synthesise the information and informing the development of a Delphi study that is accessible and engaging for service user and carer participants, The list will be reviewed by the steering committee and phrased to ensure common understanding.

### Step 2: Delphi Study and stakeholder consensus meeting

An online Delphi study will be used to reduce the lists of potential outcomes to two smaller core sets. A Delphi study is a sequential process through which the opinions of participants are sought anonymously [25]. Participants in a Delphi study do not interact directly; rather after the completion of each round of questionnaires, the collated group response is fed back to participants. In this way equal weight is given to all those who participate and the potential of an individual or group of individuals being overly influential or dominant in the process is controlled for [26].

A group of participants, representing the key stakeholder groups of service users, carers and health and social care professionals, will be recruited to the study. Those participating in the qualitative research will be invited to participate in the Delphi study. There is no consensus on the optimal sample size for a Delphi study; therefore recruitment is based on previous Delphi studies [27] and will likely result in between 75 and 100 participants being recruited to the first round of the Delphi. Participants will be purposively sampled to ensure representation of all stakeholder groups; exact numbers will be informed by previous research [27] and will be agreed upon in discussion with the steering committee and LEAP. The requirement for participants to complete all rounds of the Delphi study will be emphasised during the process of recruitment. To limit attrition appropriate procedures will be followed [27], including reminder emails.

Potential participants will be recruited from NHS trusts as well as through third sector organisations, including MIND, Bipolar UK, the McPin Foundation and NSUN. Healthcare professionals and individuals involved in research will be approached via an invitation email. Service users and carers will be recruited via email, post and leaflets and posters in NHS clinics and third sector organisations.

#### Delphi round one

In the first round participants will be asked to register online. The registration process will capture participants’ name, email addresses and additional participant information. Service users will be asked for their clinical diagnosis and the current state of health; carers will be asked for the same information of the person for which they care; healthcare professionals and commissioners will be asked for their professional role, seniority and clinical specialism. A unique identifier will be assigned to each participant to allow identification of individuals completing each round. A paper-based version of the survey will be available upon request.

The list of outcomes identified through the qualitative research and review will be presented to participants. Participants will be asked to rate the importance of each of the outcomes on a nine-point Likert scale, one being not important and nine being critical. Through the use of a free text box participants will be able to provide feedback on their choices if they wish. Through inclusion of the following question participants will be able to propose additional outcomes that were not identified in the qualitative work: ‘Can you think of any additional outcomes that should be measured in research trials with bipolar/schizophrenia service users?’ A free text box will be available for participants to list additional outcomes.

#### Round one analysis

The response rate will be assessed at the end of round one. The total number of respondents completing the round will be assessed by stakeholder groups (service users, carers and healthcare professionals). The total number of respondents will be compared to the number of respondent who agreed to participate (to analyse attrition between recruitment and survey) and the number who registered at the start of the survey (to analyse within survey attrition).

If low numbers of responders are observed in one or more of the stakeholder groups the methodology of the Delphi study will be re-assessed. Potential changes include the re-opening of round one recruitment or an additional reminder message to non-responders. If low numbers are observed in one stakeholder group only, the potential of merging groups will be assessed and discussed with the LEAP and study management team. Such decisions will be made prior to viewing results from round one to minimise bias. The following protocol is based on the assumption of adequate respondent numbers.

Data from round one will be analysed by stakeholder group. For each outcome, the number of respondents and distribution of scores will be summarised and analysed. Any additional information in a free text field will be considered by members of the study team and the LEAP to ensure they represent new outcomes not identified in previous qualitative work. If it is the case that they do represent a new outcome not already identified they will be included in round two.

#### Delphi round two

Participants from round one will be invited to participate in round two. All outcomes from round one will be carried forward into round two. The results of round one will be available for the participants in round two to review. Participants will only be able to view the results of their stakeholder group. For example, service users will be able to view the results of the service user group, but not the health and social care professional group. The number of respondents and distribution of respondent scores by clinical diagnosis will be presented.

After viewing the results of their stakeholder group from round one, participants will be asked to again rate the importance of each of the outcomes on a nine-point Likert scale. Participants will have the opportunity to review and change their scores from the previous round.

#### Delphi round two analysis

Data for round two will again be analysed by stakeholder group. For each outcome in round two, the proportion of participants scoring 1–3, 4–6 and 7–9 on the nine-point Likert scale will be calculated for each item. All outcomes will be carried forward to the next round.

#### Delphi round three

In round three participants will be presented with results of all stakeholder groups, by stakeholder group. All participants will see the scores from each stakeholder group. The proportion of participants scoring 1–3, 4–6 and 7–9 for each outcome will be presented. Participants will again be asked to use the nine-point scale to indicate whether they think the outcome should be included in the core outcome set.

#### Delphi round three analysis

Data for round three will be analysed by both the stakeholder group and all participants. For each outcome the distribution of scores will be summarised. Each outcome will be classified as “consensus in”, “consensus out” and “no consensus” using the following classification: “Consensus in”, consensus that the outcome should be included in a core outcome set, will be defined as greater than 70% of participants scoring 7–9 and less than 25% of participants scoring 1–3. “Consensus out”, consensus that the outcome should not be included in a core outcome set, will be defined as greater than 70% of participants scoring 1–3 and less than 15% scoring 7–9. “No consensus” will be said to have occurred when any other distribution of scores occurs. Such classifications are somewhat arbitrary and subjective, however stipulating the ex-ante controls for bias and prior beliefs informing the analysis post-hoc. Where “no consensus” has occurred the outcome will undergo further analysis, including assessment of the mean score in the final round. The analysis will be summarised by stakeholder group and all participants and comparison will be drawn between groups. This analysis, for both bipolar and schizophrenia, will be presented to the consensus meeting.

### Step 3: Consensus meeting

On completion of the Delphi Study a face-to-face consensus meeting will be held with key stakeholders, including some members of the LEAP. The results of each round of the Delphi study will be presented to the meeting, with the consensus results from the round three analyses used as the starting point for discussion. The final format of the meeting will be decided upon at the end of the Delphi exercise and after reviewing the experience of core outcome set projects currently in progress and drawing upon advice of COMET members.

The purpose of the meeting is to ratify the final outcome set; therefore the agenda of the meeting and processes used will be in part dependent on the consensus achieved through the Delphi study. The meeting will focus on resolving situations where “no consensus” was found to have occurred and where two outcomes classified as “consensus in” appear to assess a similar construct.

### Step 4: Systematic review of outcome measures and stakeholder meeting

The Delphi process and subsequent consensus meeting identify what core outcomes should be measured for studies involving schizophrenia and bipolar service users recruited from a community setting. A systematic review of the literature will be completed to assess the properties of existing measures used in research with bipolar and schizophrenia. Measures identified will be matched with the outcomes from the Delphi study for consideration in a later stakeholder meeting.

A systematic or rapid review [28,29] will be used to identify potential measures for inclusion in the core outcome set. This review will identify papers reporting research with people with bipolar or schizophrenia being treated in a community setting. Interventional and observational primary research will be included in the review. Measurement tools used in the identified studies will be collated and “matched” with the outcomes identified through the qualitative work and the Delphi process [30].

The measurement and psychometric properties of the measures identified will be assessed using the COSMIN checklist [31,32]. Specifically measures will be assessed for published evidence of construct validity [33], reliability [34] and responsiveness [35]. Measures that have acceptable psychometric property portfolios will be presented to the LEAP in order to assess the face validity [36] of these measures in this population.

The results of the review, assessment of the psychometric portfolios of the measures and LEAP group assessments of the measures will be presented at a stakeholder meeting. This meeting will seek consensus on recommendations of how to measure the outcomes and which measurement tools are most appropriate for use. The agenda of the meeting, and the process through which recommendations will be formed, will be designed based on the number of outcomes identified in the Delphi study and the number of corresponding measures identified in the review.

### Step 5: Stated preference survey

The key patient outcomes to be compared and explored in the stated preference survey will be defined from the evidence identified in steps 1–4 above. It may be that the work in stages 1–4 identifies one outcome and associated outcome measure that is considered the primary outcome for both people with schizophrenia and/or bipolar disorder. In this case, the stated preference survey will be designed to estimate the preferences of service users, carers and relevant healthcare professionals and policy makers for key domains of that outcome measure. However, there may be a number of outcomes that are identified as important. In this case the stated preference attributes and levels will be developed from the results of stage 1–4 and refined by discussions with the LEAP and study team. LEAP participants will also be asked to participate in a pilot of the survey measure.

Service users, carers and relevant health and social care professionals and commissioners will be asked to complete the stated preference survey to identify their preferences and priorities for the different types of outcomes identified as important to measure in the core set. The participants will be drawn from the focus groups and Delphi panels.

The stated preference survey will use orthogonal main effects. Responses from the questionnaires will be analysed using appropriate logistic or probit regression analyses. The coefficients for each attribute will indicate the direction of preference for that attribute. Marginal rates of substitution will be calculated to estimate the relative utility of the attribute. These analyses will be used to:

1. explore the relative importance and preference for different aspects of outcome included in the core set (or primary outcome) and
2. estimate preference weights that can be used to combine key domains into a single index.

## Discussion/conclusion

A core outcome set represents the minimum measurement requirement for a research area. Studies within that area which are focussed on a sub-set of service users, for example rapid cycling bipolar, or focussed on specific area, for example collaborative care, may feel the requirement to supplement the a core outcome set with additional, relevant measures assessing different outcomes.

This protocol has a number of strengths, including the commitment to ensuring service users and carers are represented, using qualitative work to identify outcomes that may not currently be used in research and following best practice developed through the COMET initiative. Some potential limitations of this work should be highlighted. The requirement of service users and carers to self-identify could induce bias or inaccuracy. The use of an online Delphi survey will limit participation to those who are computer literate and the qualitative work cannot include those who do not have a conversational level of spoken English. Reasonable steps will be taken to minimise the impact of these limitations upon the work and they will be reflected upon when presenting findings.

There are no core outcome sets for use in research involving service users with schizophrenia or bipolar managed in a community setting. The PARTNERS2 project aims to develop core outcome sets for these research areas by drawing on outcomes identified as important by relevant stakeholders and using the expertise of our LEAP. Given that 94% of diagnoses for SMI in primary care were for bipolar or schizophrenia we anticipate that it is likely that there will be some degree of convergence between the two sets developed [2]. The potential of developing one core outcome set for use in SMI research in a community setting will be continually assessed throughout this work. It is anticipated that successful completion of this work will improve the ability of future research to draw comparison between studies involving people with schizophrenia and bipolar and improve interpretation of results. The challenges of developing a COS in this area and with this population will be discussed and subsequent weaknesses of the research will be highlighted.

## Trial status

At the time of manuscript submission the status of the trial is ongoing. Patient recruitment has not been completed.

## Endnotes

^a^Where the term outcome or outcomes is used this refers to an outcome domain (for example, quality of life or physical functioning). The term outcome measure will be used to refer to measurement tools.

^b^Participants will be encouraged to take part in focus groups; however some participants may feel unable to participate in such groups. Reasons could be practical (e.g. they cannot attend on the date of the focus group) or more complex (e.g. the format of a focus group is intimidating for the participant). In these situations the potential participant will be offered the opportunity to take part in a one-to-one interview.

^c^These participants will be referred to as “health and social care professionals” in the remainder of this protocol.

## Abbreviations

COMET: Core Outcome Measurement in Effectiveness Trials
COSMIN: COnsensus-based Standards for the selection of health Measurement INstruments
CRN: Clinical research network
LEAP: Lived Experience Advisory Panel
NSUN: National Survivor User Network
PROM: Patient reported outcome measure
RCT: Randomised controlled trials
SMI: serious mental illness.
